# Interplay between sugar and hormone signaling pathways modulate floral signal transduction

**DOI:** 10.3389/fgene.2014.00218

**Published:** 2014-08-13

**Authors:** Ianis G. Matsoukas

**Affiliations:** ^1^Institute for Renewable Energy and Environmental Technologies, University of BoltonBolton, UK; ^2^Systems and Synthetic Biology, Institute for Materials Research and Innovation, University of BoltonBolton, UK

**Keywords:** *Arabidopsis thaliana*, florigenic and antiflorigenic signaling, juvenile-to-adult phase transition, juvenility, signal transduction, sugar-hormone interactions, vegetative-to-reproductive phase transition

## Abstract

**NOMENCLATURE** The following nomenclature will be used in this article:

- Names of genes are written in italicized upper-case letters, e.g., *ABI4*.
- Names of proteins are written in non-italicized upper-case letters, e.g., ABI4.
- Names of mutants are written in italicized lower-case letters, e.g., *abi4*.

The juvenile-to-adult and vegetative-to-reproductive phase transitions are major determinants of plant reproductive success and adaptation to the local environment. Understanding the intricate molecular genetic and physiological machinery by which environment regulates juvenility and floral signal transduction has significant scientific and economic implications. Sugars are recognized as important regulatory molecules that regulate cellular activity at multiple levels, from transcription and translation to protein stability and activity. Molecular genetic and physiological approaches have demonstrated different aspects of carbohydrate involvement and its interactions with other signal transduction pathways in regulation of the juvenile-to-adult and vegetative-to-reproductive phase transitions. Sugars regulate juvenility and floral signal transduction through their function as energy sources, osmotic regulators and signaling molecules. Interestingly, sugar signaling has been shown to involve extensive connections with phytohormone signaling. This includes interactions with phytohormones that are also important for the orchestration of developmental phase transitions, including gibberellins, abscisic acid, ethylene, and brassinosteroids. This article highlights the potential roles of sugar-hormone interactions in regulation of floral signal transduction, with particular emphasis on *Arabidopsis thaliana* mutant phenotypes, and suggests possible directions for future research.

## Introduction

The greatest advances in our understanding of the genetic regulation of developmental transitions have derived from studying the vegetative-to-reproductive phase transition in several dicot and monocot species. This has led to the elucidation of multiple environmental and endogenous pathways that promote, enable and repress floral induction (reviewed in Matsoukas et al., 2012). The photoperiodic (Kardailsky et al., 1999; Kobayashi et al., 1999) and vernalization (Schmitz et al., 2008) pathways regulate time to flowering in response to environmental signals such as daylength, light and temperature, whereas the autonomous (Jeong et al., 2009), aging (Yang et al., 2013; Yu et al., 2013) and gibberellin (GA)-dependent (Porri et al., 2012) pathways monitor endogenous indicators of the plant's age and physiological status. In addition, other factors and less characterized pathways also play a role in regulation of floral signal transduction. These include ethylene (Achard et al., 2006), brassinosteroids (BRs; Domagalska et al., 2010), salicylic acid (Jin et al., 2008) and cytokinins (D'aloia et al., 2011).

The photoperiodic pathway is probably the most conserved of the floral induction pathways. It is known for its promotive effect by relaying light and photoperiodic timing signals to floral induction (reviewed in Matsoukas et al., 2012). This pathway involves genes such as *PHYTOCHROMES* (*PHYs*; Sharrock and Quail, 1989; Clack et al., 1994) and *CRYPTOCHROMES* (*CRYs*; Ahmad and Cashmore, 1993; Guo et al., 1998; Kleine et al., 2003), which are involved in the regulation of light signal inputs. Other genes such as *GIGANTEA* (*GI*; Fowler et al., 1999), *CIRCADIAN CLOCK ASSOCIATED1* (*CCA1*; Wang et al., 1997), and *LATE ELONGATED HYPOCOTYL* (*LHY*; Schaffer et al., 1998) are components of the circadian clock, whereas *CONSTANS* (*CO*), *FLOWERING LOCUS T* (*FT*; Kardailsky et al., 1999; Kobayashi et al., 1999), *TWIN SISTER OF FT* (*TSF*; Yamaguchi et al., 2005), and *FLOWERING LOCUS D* (*FD*; Abe et al., 2005) encode proteins that specifically regulate floral induction. The actions of all pathways ultimately converge to control the expression of so-called floral pathway integrators (FPIs), which include *FT* (Corbesier et al., 2007), *TSF* (Yamaguchi et al., 2005), *SUPPRESSOR OF CONSTANS1* (*SOC1*; Yoo et al., 2005), and *AGAMOUS-LIKE24* (*AGL24*; Lee et al., 2008; Liu et al., 2008). These act on floral meristem identity (FMI) genes *LEAFY* (*LFY*; Lee et al., 2008), *FRUITFUL* (*FUL*; Melzer et al., 2008), and *APETALA1* (*AP1*; Wigge et al., 2005; Yamaguchi et al., 2005), which result in floral initiation. On the other hand, pathways that enable floral induction regulate the expression of floral repressors or translocatable florigen antagonists, known as antiflorigens (reviewed in Matsoukas et al., 2012). The pathways that regulate the floral repressor *FLOWERING LOCUS C* (*FLC*) are the best-characterized (reviewed in Michaels, 2009).

The vegetative-to-reproductive phase transition is preceded by the juvenile-to-adult phase transition within the vegetative phase (reviewed in Poethig, 1990, 2013; Matsoukas et al., 2013; Matsoukas, 2014). During the juvenile phase plants are incapable of initiating reproductive development and are insensitive to environmental stimuli such as photoperiod and vernalization, which induce flowering in adult plants (Matsoukas et al., 2013; Matsoukas, 2014; Sgamma et al., 2014). The juvenile-to-adult phase transition is accompanied by a decrease in microRNA156 (miR156A/miR156C) abundance and a concomitant increase in abundance of miR172, as well as the *SQUAMOSA PROMOTER BINDING PROTEIN-LIKE* (*SPL3/4/5*) transcription factors (TFs; Wang et al., 2009; Wu et al., 2009; Jung et al., 2011, 2012; Kim et al., 2012). Expression of miR172 activates *FT* transcription in leaves through repression of AP2-like transcripts *SCHLAFMÜTZE* (*SMZ*), *SCHNARCHZAPFEN* (*SNZ*), and *TARGET OF EAT 1-3* (*TOE1-3*; Jung et al., 2007, 2011; Mathieu et al., 2009), whereas the increase in *SPLs* at the shoot apical meristem (SAM), leads to the transcription of FMI genes (Schwab et al., 2005; Schwarz et al., 2008; Wang et al., 2009; Yamaguchi et al., 2009). Therefore, from a molecular perspective juvenility can be defined as the period during which the abundance of antiflorigenic signals such as miR156/miR157 is sufficiently high to repress the transcription of *FT* and *SPL* genes (Matsoukas, 2014).

Carbohydrates serve diverse functions in plants ranging from energy sources, osmotic regulators, storage molecules, and structural components to intermediates for the synthesis of other organic molecules (reviewed in Rolland et al., 2006; Smeekens et al., 2010; Eveland and Jackson, 2012). Carbohydrates also act as signaling molecules (Moore et al., 2003) and by their interaction with mineral networks (Zakhleniuk et al., 2001; Lloyd and Zakhleniuk, 2004) affect the juvenile-to-adult and vegetative-to-reproductive phase transitions (Matsoukas et al., 2013). Interestingly, sugar signaling has been shown to involve extensive interaction with hormone signaling (Zhou et al., 1998; Arenas-Huertero et al., 2000; Moore et al., 2003). This includes interactions with hormones that are also important for the regulation of juvenile-to-adult and vegetative-to-reproductive phase transitions, including GAs (Yuan and Wysocka-Diller, 2006), abscisic acid (ABA; Arenas-Huertero et al., 2000; Laby et al., 2000), ethylene (Zhou et al., 1998), and BRs (Goetz et al., 2000; Schluter et al., 2002). Several molecular mechanisms that mediate sugar responses have been identified in plants (reviewed in Rolland et al., 2006; Smeekens et al., 2010). The best examples involve hexokinase (HXK; Moore et al., 2003), trehalose-6-phosphate (Tre6P; Van Dijken et al., 2004) and the sucrose non-fermenting 1-related protein kinase1 (SnRK1; Baena-Gonzalez et al., 2007) complex. SnRK1 has a role when sugars are in extremely limited supply, whereas HXK and Tre6P play a role in the presence of excess sugar.

The panoptic themes of floral signal transduction, sugar sensing and signaling, and hormonal regulation of growth and development have attracted much attention, and many comprehensive review articles have been published (Rolland et al., 2006; Amasino, 2010; Smeekens et al., 2010; Depuydt and Hardtke, 2011; Huijser and Schmid, 2011; Andres and Coupland, 2012). This article, however, focuses specifically on sugar-hormone interactions and their involvement in regulation of floral signal transduction, with particular emphasis on *Arabidopsis thaliana* mutant phenotypes. The review is divided into two sections: the first provides several pieces of evidence on the interactions between sugars and different hormones in floral induction; whereas the second describes potential mechanisms that might be involved in regulation of floral signal transduction, in response to sugar-hormone interplay.

## Sugar/hormone interactions and floral signal transduction

### The sugar and gibberellin signaling crosstalk

GAs are a group of molecules with a tetracyclic diterpenoid structure that function as plant growth regulators influencing a range of developmental processes. Several *Arabidopsis* mutants in the GA signal transduction and GA biosynthesis pathway have been isolated (Table 1; Peng and Harberd, 1993; Peng et al., 1997; Hedden and Phillips, 2000). Null mutations in the early steps of GA biosynthesis (e.g., *ga1-3*) do not flower in short days (SDs), whereas weak mutants (e.g., *ga1-6*; Koornneef and Van Der Veen, 1980), or GA signal transduction mutants [e.g., *gibberellic acid insensitive* (*gai*)], flower later than wild type (WT; Peng and Harberd, 1993). In contrast, mutants with increased GA signaling such as *rga like2* (*rgl2*; Cheng et al., 2004; Yu et al., 2004) and *spindly* (*spy*; Jacobsen and Olszewski, 1993) have an early flowering phenotype. Evidence has been provided that both *RGL2* and *SPY* might be involved in carbohydrate regulation of floral initiation, as mutation in both *loci* confers insensitivity to inhibiting glucose concentrations (Yuan and Wysocka-Diller, 2006). SPY, an O-linked *B*-N-acetylglucosamine transferase was shown to interact with the GI in yeast (Tseng et al., 2004). Mutants impaired in *GI* have a late flowering and starch-excess phenotype (Eimert et al., 1995). The interaction between SPY and GI suggests that functions of these proteins might be related, and that *SPY* might be a pleiotropic circadian clock regulator (Tseng et al., 2004; Penfield and Hall, 2009). In addition, the early flowering phenotype of the glucose insensitive *spy* may be *via FT*, as *spy4* suppresses the reduction of *CO* and *FT* mRNA in *gi2* genotypes (Tseng et al., 2004). This indicates that *SPY* functions in the photoperiod pathway upstream of *CO* and *FT*, involving glucose and GA metabolism-related events. Interestingly, it has been suggested that *SPY4* may play a central role in the regulation of GA/cytokinin crosstalk during plant development (Greenboim-Wainberg et al., 2005).

**Table 1:** **List of genes in Arabidopsis *thaliana* that regulate floral signal transduction in response to sugar-hormone interplay**.

**Gene name**	**Abbreviation**	**Allelic**	**Gene identifier**	**Description**	**Flowering mutant phenotype**^a^		**References**
					**SD**	**LD**	
**SUGAR-GA SIGNALING CROSSTALK**							
*GA REQUIRING 1-3*	*GA1-3*	*CPS, KSA*	At4g02780	GA biosynthesis; ent-copalyl diphosphate synthase/magnesium ion binding	No phenotype	No phenotype	Koornneef and Van Der Veen, 1980
*GA REQUIRING 1-6*	*GA1-6*	*CPS, KSA*	At4g02780	GA biosynthesis	Late	Late	Koornneef and Van Der Veen, 1980
*GIBBERELLIC ACID INSENSITIVE*	*GAI*	*GRAS-3, RGA2*	At1g14920	TF^b^; repressor of GA responses; involved in GA mediated signaling	Late	Late	Peng and Harberd, 1993; Peng et al., 1997; Hedden and Phillips, 2000
*RGA LIKE 2*	*RGL 2*	*GRAS-15, SCL19, DELLA protein RGL2*	At3g03450	TF; SCARECROW-like; GA signaling; encodes a DELLA protein	Early	Early	Cheng et al., 2004; Tyler et al., 2004; Yu et al., 2004
*SPINDLY*	*SPY*	n/a	At3g11540	Repressor of GA responses; positive regulator of cytokinin signaling; glucose insensitive mutant	Early	Early	Jacobsen and Olszewski, 1993; Swain et al., 2002; Greenboim-Wainberg et al., 2005
*GIGANTEA*	*GI*	n/a	At1g22770	Starch excess mutant; component of the circadian oscillator	Late	Similar or later than WT	Eimert et al., 1995; Tseng et al., 2004; Penfield and Hall, 2009
*LEAFY*	*LFY*	*MAC9_13*	At5g61850	TF; sugar and GA regulated	No phenotype	No phenotype	Blazquez et al., 1998; Eriksson et al., 2006
**SUGAR-ABA SIGNALING CROSSTALK**							
*ABA DEFICIENT 2*	*ABA2*	*GIN1, ISI4, SAN3, SDR1, SIS4, SRE1*	At1g52340	Oxidoreductase; molecular link between sugar signaling and hormone biosynthesis	Early	Early	Laby et al., 2000; Rook et al., 2001; Cheng et al., 2002
*ABA DEFICIENT 3*	*ABA3*	*GIN5, ISI2, SIS3*	At1g16540	Involved in the conversion of ABA-aldehyde to ABA; glucose insensitive mutant; mo-molybdopterin cofactor sulfurase	Early	Early	Leon-Kloosterziel et al., 1996; Arenas-Huertero et al., 2000; Bittner et al., 2001
*ABA INSENSITIVE 3*	*ABI3*	*SIS10*	At3g24650	TF; molecular link between sugar signaling and hormone biosynthesis	Early	Early	Giraudat et al., 1992; Huang et al., 2008
*ABA INSENSITIVE 4*	*ABI4*	*GIN6, ISI3, SIS5, SUN6*	At2g40220	TF; molecular link between sugar signaling and hormone biosynthesis	Similar or slightly earlier than WT	Similar to WT	Finkelstein et al., 1998; Arenas-Huertero et al., 2000; Matsoukas et al., 2013
*CIRCADIAN CLOCK ASSOCIATED 1*	*CCA1*	*MYB-RELATED DNA BINDING PROTEIN*	At2g46830	TF; component of the circadian oscillator	Early	Similar to WT	Mizoguchi et al., 2002; Hanano et al., 2006
*TIMING OF CAB EXPRESSION 1*	*TOC1*	*ABI3 INTERACTING PROTEIN 1, PRR1*	At5g61380	TF; contributes to the plant fitness (carbon fixation, biomass) by influencing the circadian oscillator period	Early	Early	Kreps and Simon, 1997; Somers et al., 1998; Kurup et al., 2000; Pokhilko et al., 2013
**SUGAR-ETHYLENE SIGNALING CROSSTALK**							
*CONSTITUTIVE TRIPLE RESPONSE1*	*CTR1*	*GIN4, SIS1*	At5g03730	Kinase; negative regulator of ethylene signaling; sugar signaling	Late	Late	Gibson et al., 2001; Cheng et al., 2002; Achard et al., 2007
*ETHYLENE INSENSITIVE 2*	*EIN2*	*CKR1, ERA3*	At5g03280	Transporter; involved in ethylene signal transduction	Late	Late	Su and Howell, 1992; Fujita and Syono, 1996; Zhou et al., 1998; Alonso et al., 1999
*ETHYLENE OVERPRODUCER 1*	*ETO1*	n/a	At3g51770	Protein binding; promote ethylene biosynthesis	Early	Early	Bleecker et al., 1988; Guzman and Ecker, 1990; Roman et al., 1995; Chae et al., 2003
*ETHYLENE RESPONSE 1*	*ETR1*	*EIN1*	At1g66340	Ethylene binding; ethylene receptor; protein histidine kinase	Late	Late	Bleecker et al., 1988; Guzman and Ecker, 1990; Chang et al., 1993; Chen and Bleecker, 1995
*ETHYLENE RESPONSE 2*	*ETR2*	n/a	At3g23150	Negative regulation of ethylene mediated signaling pathway; glycogen synthase kinase3; protein histidine kinase	Early	Similar or slightly later than WT	Sakai et al., 1998
**SUGAR-BR SIGNALING CROSSTALK**							
*BRASSINOSTEROID, LIGHT AND SUGAR 1*	*BLS1*	n/a	n/a^c^	Component for BR and light responsiveness; involved in sugar signaling	Late	Late	Laxmi et al., 2004
*CONSTITUTIVE PHOTOMORPHOGENESIS AND DWARFISM*	*CPD*	*CBB3, CYP90, DWARF3*	At5g05690	Electron carrier; heme binding; iron ion binding; monooxygenase; oxygen binding; under circadian and light control	Late	Late	Szekeres et al., 1996; Li and Chory, 1997; Choe et al., 1998; Domagalska et al., 2007
*DE-ETIOLATED 2*	*DET2*	*DWARF6*	At2g38050	Similar to mammalian steroid-5-alpha-reductase; involved in the brassinolide biosynthetic pathway	Late	Late	Li et al., 1996; Noguchi et al., 1999; Tanaka et al., 2005

a *The flowering mutant phenotype compared to WT, under short (SD; 8 h light) and long day (LD; 16 h light) conditions*.

b *TF, transcription factor*.

c *The mutation has been mapped within a 1.4 Mb region of chromosome 5 (Laxmi et al., 2004)*.

Lines of evidence have demonstrated that there is a synergistic interaction between GAs and sucrose in the activation of *LFY* transcription (Blazquez et al., 1998; Eriksson et al., 2006). These pieces of evidence suggest a further link between GAs with sugar metabolism-related events and floral signal transduction. The effects of GA-sugar interplay on regulation of floral induction might be transduced by the *GIBBERELLIN INSENSITIVE DWARF1* (*GID1*), which act upstream of the DELLA (Feng et al., 2008; Harberd et al., 2009), and *PHYTOCHROME-INTERACTING FACTOR* (*PIF*; De Lucas et al., 2008; Nozue et al., 2011; Stewart et al., 2011) family of bHLH factors.

### The sugar-ABA signaling crosstalk

ABA is regarded as a general inhibitor of floral induction. This is indicated in *Arabidopsis* where mutants deficient (e.g., *aba2, aba3*) in or insensitive [e.g., *aba insensitive4* (*abi4*)] to ABA are early flowering (Table 1; Martinez-Zapater et al., 1994). On the other hand, mutants with high ABA levels [e.g., *no hydrotropic response* (*nhr1*)] flower late or even later than WT under non-inductive SDs (Quiroz-Figueroa et al., 2010). However, many mutations affecting sugar signaling are allelic with components of the ABA synthesis or ABA transduction pathways. It has been shown that *aba2, aba3*, and *abi4* mutants are allelic to sugar-insensitive mutants *glucose insensitive1* (*gin1*)*/impaired sucrose induction4* (*isi4*)*/sugar insensitive1* (*sis1*; Laby et al., 2000; Rook et al., 2001), *gin5/isi2/sis3* (Arenas-Huertero et al., 2000) and *gin6/isi3/sis5/sun6* (Arenas-Huertero et al., 2000), respectively. In addition, ABA accumulation and transcript levels of several ABA biosynthetic genes are significantly increased by glucose (Cheng et al., 2002). These lines of evidence indicate that signaling pathways mediated by ABA and sugars may interact to regulate juvenility and floral signal transduction (Matsoukas et al., 2013).

The downstream effects of the sugar-ABA interaction might be mediated *via* the circadian clock. Photoperiodic induction requires the circadian clock to measure the duration of the day or night (reviewed in Harmer, 2009; Imaizumi, 2010). The clock modulates the expression of *CO*, the precursor of *FT* that accelerates flowering in response to several pathways (reviewed in Turck et al., 2008). It has been shown that glucose has a marked effect on the entrainment and maintenance of robust circadian rhythms (Dalchau et al., 2011; Haydon et al., 2013). In addition, circadian periodicity is also regulated by ABA *via* an unclear mechanism. This might be through *ABI3* (allelic to *sis10*; Huang et al., 2008) by binding to the clock component *TIMING OF CAB EXPRESSION1* (*TOC1*; also called ABI3 Interacting Protein 1; Kurup et al., 2000; Pokhilko et al., 2013), and/or regulation of *CCA1* mRNA transcription levels by ABA (Hanano et al., 2006). Thus, gating of circadian clock sensitivity by the ABA and sugar crosstalk may constitute a regulatory module that coordinates the circadian clock with additional endogenous and environmental signals to regulate juvenility and floral signal transduction.

### The sugar-ethylene signaling crosstalk

Ethylene is another example of a phytohormone that regulates juvenility (Beyer and Morgan, 1971) and floral induction (Bleecker et al., 1988; Guzman and Ecker, 1990). *Arabidopsis* mutants impaired in ethylene signaling [e.g., *ethylene insensitive2* (*ein2*), *ein3-1*] or perception [e.g., *ethylene response1* (*etr1-1*)], flower late in inductive LDs (Table 1). This late flowering phenotype is significantly enhanced under non-inductive SDs. Mutants, which over-produce ethylene [e.g., *ethylene overproducer1* (*eto1*), *eto2-1*] flower at the same time or slightly earlier than WT under LDs, but dramatically later in SDs (Bleecker et al., 1988; Guzman and Ecker, 1990; Chen and Bleecker, 1995; Achard et al., 2007). Ample evidence has shown that ethylene can influence plant sensitivity to sugars. Ethylene-insensitive plants are more sensitive to endogenous glucose, whereas application of an ethylene precursor decreases glucose sensitivity (Zhou et al., 1998; Leon and Sheen, 2003). However, this interaction may also function in an antithetical way as several ethylene biosynthetic and signal transduction genes are repressed by glucose (Yanagisawa et al., 2003; Price et al., 2004).

Ethylene sensing and signaling pathways are also tightly interconnected with those for sugar and ABA (reviewed in Gazzarrini and Mccourt, 2001; Leon and Sheen, 2003). Lines of evidence have shown that this crosstalk modulates the vegetative-to-reproductive phase transition. This is suggested by the glucose hypersensitive phenotype displayed by the late flowering mutants *ein2* [allelic to *enhanced response to aba3* (*era3*)], *ein3* and *etr1* (Chang et al., 1993; Zhou et al., 1998; Alonso et al., 1999; Cheng et al., 2002; Yanagisawa et al., 2003). Activation of the ethylene response [either in the presence of exogenous ethylene or by means of the *eto1* or *constitutive triple response1* (*ctr1*) mutations] attenuates the glucose effects (Zhou et al., 1998; Gibson et al., 2001). Further support for the sugar-ethylene crosstalk involvement on flowering time is derived by the epistatic analysis of the *etr1 gin1* (*aba2*) and *ein2 gin1* (*aba2*) double mutants in the elucidated role of *GIN1* (*ABA2*) in the ethylene signal transduction cascade. The *etr1 gin1* (*aba2*) and *ein2 gin1* (*aba2*) double mutants flower earlier than *etr1* and *ein2* single mutants, respectively (Cheng et al., 2002). The early flowering and glucose resistance phenotypes of the double mutants *etr1 gin1* (*aba2*) and *ein2 gin1* (*aba2*) under LDs, may suggest that ethylene affects glucose signaling, partially, through ABA to regulate floral induction (Zhou et al., 1998; Cheng et al., 2002; Ghassemian et al., 2006). Overexpression of *ETHYLENE RESPONSE2* (*ETR2*; Sakai et al., 1998) receptor in *Oryza sativa* reduced ethylene sensitivity and delayed floral induction (Wuriyanghan et al., 2009). Conversely, disruption of *ETR2* by T-DNA or with RNA interference (RNAi) conferred enhanced ethylene sensitivity and early flowering. Moreover, links of the ethylene signaling with starch accumulation responses and activation of sugar transporter genes have also been observed. *ETR2* promoted starch accumulation, whereas a monosaccharide transporter gene was suppressed in the *ETR2* over-expression lines (Wuriyanghan et al., 2009). Interestingly, when expression of *ETR2* was reduced in the *OSetr2* T-DNA and RNAi lines, starch failed to accumulate, whereas sugar translocation was enhanced (Wuriyanghan et al., 2009).

Ethylene has dramatic effects on flowering time of mutants involved in activation of the ethylene response under SD conditions (Achard et al., 2007). *CONSTITUTIVE TRIPLE RESPONSE1* (*CTR1*) is a major negative regulator of ethylene signaling that is allelic to *GIN4* (Cheng et al., 2002) and *SIS1* (Gibson et al., 2001). Loss-of-function *ctr1* mutations result in the constitutive activation of the ethylene response pathway, which indicates that the encoded protein acts as a negative regulator of ethylene signaling (Kieber et al., 1993). Under LDs *ctr1* has a flowering phenotype similar to WT. In antithesis with the other glucose insensitive genotypes, *ctr1* plants flower dramatically later than WT in SDs. This could be due to impaired involvement of GA pathway, which systematize floral initiation in SDs. Interestingly, evidence has been provided that ethylene dramatically prolongs time to flowering in *ctr1* under SDs by repressing the up-regulation of *LFY* and *SOC1* transcript levels *via* a DELLA-dependent mechanism, and decreasing the levels of the endogenous bioactive GAs (Achard et al., 2007).

### The sugar-brassinosteroids signaling crosstalk

BRs are steroid hormones known to control various skotomorphogenic (Chory et al., 1991) and photomorphogenic (Li et al., 1996) aspects of development. Genetic and physiological analyses have revealed the critical role of BRs in floral induction (Table 1), establishing a new floral signal transduction pathway. The promotive role of BRs on floral induction is exerted by the late flowering phenotype of BR-deficient mutants *brassinosteroid-insensitive1* (*brs1*; Clouse et al., 1996; Li and Chory, 1997), *brassinosteroid-insensitive2* (*bin2*; Li et al., 2001), *deetiolated2* (*det2*; Chory et al., 1991), *constitutive photomorphogenesis and dwarfism* (*cpd*; Szekeres et al., 1996; Domagalska et al., 2007) and *brassinosteroid, light and sugar1* (*bls1*; Laxmi et al., 2004). Conversely, mutations impaired in metabolizing BRs to their inactive forms, *phyB-activation-tagged suppressor1* (*bas1*; Neff et al., 1999) and *suppressor of phyB-4 7* (*sob7*; Turk et al., 2005) flower early (Turk et al., 2005). It has been reported that the response to exogenously applied BRs differs depending on the light quality and quantity (Neff et al., 1999), suggesting a potential interaction with sugars *via* light-mediated pathways (Goetz et al., 2000; Schluter et al., 2002). In addition, it has been demonstrated that BR responses are related to hormones such as GA (Gallego-Bartolome et al., 2012), ABA (Domagalska et al., 2010), and ethylene (Turk et al., 2005), which participate in sugar signaling. Furthermore, the sugar hypersensitive phenotype of the late flowering *bls1* can be repressed by exogenous BRs (Laxmi et al., 2004). Moreover, the late flowering mutant *det2*, as other constitutively photomorphogenic mutants have been found to have an altered response to applied sugars (reviewed in Chory et al., 1996; Laxmi et al., 2004, and references therein). Collectively, these data indicate interplay between BRs and sugars in regulation of floral signal transduction. The downstream effects of this crosstalk might be mediated through *BRASSINAZOLE RESISTANT1* (*BZR1*) and *BZR2*, as well as additional interacting TFs. Both BZR1 and BZR2 interact with PIF (Oh et al., 2012) and the GA signaling DELLA proteins (Oh et al., 2012). In addition, the BR-sugar interaction may also be indirectly involved in modulation of juvenility and floral signal transduction by influencing the photoperiodic pathway *via* the circadian clock, as BR application shortens circadian rhythms (Hanano et al., 2006).

## How does the crosstalk between sugars and hormones regulate the floral signal transduction

**Figure 1:**
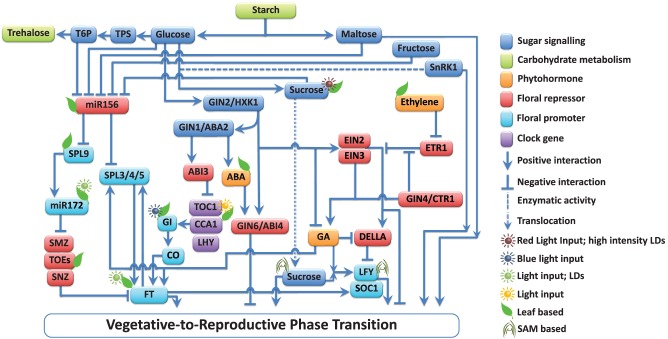
**Multiple interactions among the components involved in floral signal transduction in response to sugar-hormone interplay**. Components of the pathways are grouped into those that promote (↓) and those that repress (⊥) floral signal transduction. Sugars affect the vegetative-to-reproductive phase transition through their function as energy sources, osmotic regulators, signaling molecules, and by their interaction with mineral and phytohormone networks (Ohto et al., 2001; Lloyd and Zakhleniuk, 2004; Matsoukas et al., 2013). Starch metabolism-related events have a key role in developmental phase transitions (Corbesier et al., 1998; Matsoukas et al., 2013). The actions of all pathways ultimately converge to control the expression of a small number of so-called floral pathway integrators (FPIs), which include *FLOWERING LOCUS T* (*FT*; Kardailsky et al., 1999; Kobayashi et al., 1999) and *SUPPRESSOR OF CONSTANS1* (*SOC1*; Yoo et al., 2005). These act on floral meristem identity (FMI) genes such as *LEAFY* (*LFY*; Lee et al., 2008) and *APETALA1* (*AP1*; Wigge et al., 2005; Yamaguchi et al., 2005), which result in floral induction. The main components and interactions are depicted in the diagram, but additional elements have been omitted for clarity. Comprehensive reviews are available (Smeekens et al., 2010; Depuydt and Hardtke, 2011; Huijser and Schmid, 2011; Andres and Coupland, 2012; Matsoukas et al., 2012) and should be referred to for additional pieces of information.

It is proposed that the effects of the sugar-hormone interplay might be mediated by hormones that enable tissues to respond to sugars, and/or hormone and sugar signaling, although essentially separate, could converge and crosstalk through specific regulatory complexes (Figure 1). One regulatory mechanism might be through metabolic enzymes, which also function as active members of transcriptional or posttranscriptional regulatory complexes (Cho et al., 2006). This cross-functionalization could be involved in mechanisms that modulate juvenility and floral signal transduction, by allowing interplay between different sugar and hormone response pathways or receptors.

### The HXK1-miR156 regulatory module

Sugar signals can be generated either by carbohydrate concentration and relative ratios to other metabolites, such as hormones (Arenas-Huertero et al., 2000) and carbon-nitrogen ratio (Corbesier et al., 2002; Rolland et al., 2006), or by flux through sugar-specific transporters (Lalonde et al., 1999) and/or sensors (Moore et al., 2003). Sugar sensors perceive the presence of different sugars and initiate downstream signaling events. Glucose (Moore et al., 2003), fructose (Cho and Yoo, 2011; Li et al., 2011), sucrose (Seo et al., 2011), Tre6P (Van Dijken et al., 2004), and maltose (Niittyla et al., 2004; Stettler et al., 2009) function as cellular signaling molecules in specific regulatory pathways, which modulate juvenility and floral signal transduction. Of these signaling molecules, glucose has been studied the most comprehensively in plants.

Glucose-mediated floral signal transduction is largely dependent on HXK, HXK-independent, and SnRK1 signaling pathways. One possibility is that HXK1 controls juvenility and floral signal transduction by regulating the expression of miR156 (Yang et al., 2013). In this scenario, HXK1 that is largely dependent on ABA biosynthesis and signaling components (Zhou et al., 1998; Arenas-Huertero et al., 2000) promotes miR156 expression under low sugar levels. Above a threshold concentration, the circadian fluctuations of glucose, one of the final outputs of starch degradation (Stitt and Zeeman, 2012) that is regulated by starch and Tre6P (Martins et al., 2013) promotes GA biosynthesis (Cheng et al., 2002; Yu et al., 2012; Paparelli et al., 2013) and blocks HXK1 activity, resulting in downregulation of miR156 expression (Yang et al., 2013; Yu et al., 2013). Interestingly, defoliation experiments (Yang et al., 2011, 2013; Yu et al., 2013) show that removing the two oldest leaves results in increased miR156 levels at the SAM and a prolonged juvenile phase length. The fact that glucose, fructose, sucrose and maltose, partially, reverse this effect (Wang et al., 2013; Yu et al., 2013), indicates that photosynthetically derived sugars are potential components of the signal transduction pathway that repress miR156 expression in leaf primordia.

It seems highly probable that the differential regulation of SnRK1 by ABA and GAs (Bradford et al., 2003), and the antagonism between ABA and GA, which function in an opposite manner, to activate specific *cis*-acting regulatory elements present in ABA- and GA-responsive promoters respectively (reviewed in Yamaguchi-Shinozaki and Shinozaki, 2005), may also be involved in this regulatory module (Achard et al., 2006; Yu et al., 2012; Wang et al., 2013).

### The Tre6P-miR156 regulatory module

Tre6P is a metabolite of emerging significance in plant developmental biology, with hormone-like metabolic activities (reviewed in Smeekens et al., 2010; Ponnu et al., 2011). It has been proposed that Tre6P signals the availability of sucrose (Lunn et al., 2006), and then through the SnRK1 regulatory system orchestrates changes in gene expression that enable sucrose to regulate juvenility and floral signal transduction. In *Arabidopsis*, Tre6P is synthesized from glucose-6-phospate by *TREHALOSE PHOSPHATE SYNTHASE 1* (*TPS1*; Van Dijken et al., 2004). Non-embryo-lethal weak alleles of *tps1* exhibit late flowering (Van Dijken et al., 2004) and ABA hypersensitive phenotypes (Gomez et al., 2010). Interestingly, the Tre6P pathway controls the expression of *SPL3, SPL4*, and *SPL5* at the SAM, partially *via* miR156, and partly independently of the miR156-dependent pathway *via FT* (Wahl et al., 2013). Several pieces of evidence suggest that Tre6P inhibits SnRK1 when sucrose is above a threshold concentration (Polge and Thomas, 2007; Zhang et al., 2009). When the sucrose content decreases, with Tre6P decreasing as well, SnRK1 is released from repression, which leads to the induction of genes involved in photosynthesis-related events, so that more carbon is made available (Delatte et al., 2011). It has been shown that the Tre6P-SnRK1 module acts through a mechanism involving ABA (Gomez et al., 2010) and sugar metabolism (Van Dijken et al., 2004) to regulate several developmental events. The key link between sugars and ABA perception is exemplified by the *ABI* genes (Eveland and Jackson, 2012; Wang et al., 2013). Interestingly, *ABI4* encodes an AP2 domain TF that is required for normal sugar responses during the early stages of development (Arenas-Huertero et al., 2000; Laby et al., 2000; Rook et al., 2001; Niu et al., 2002). Taken together, these data could provide another mechanistic link, at the molecular level, on how the ABA-sugar interplay might be involved in regulation of juvenility and floral signal transduction.

## Perspectives

Sugars serve diverse functions in plants ranging from energy sources, osmotic regulators, storage molecules, and structural components to intermediates for the synthesis of other organic molecules. Sugars also act as signaling molecules and by their interaction with mineral and hormonal networks affect several aspects of growth and development.

There has been a long-standing interest in the role played by sugars and hormones in regulation of the juvenile-to-adult and vegetative-to-reproductive phase transitions. It has been proposed that the effects of sugar-hormone interactions might be mediated by key hormones that enable tissues to respond to sugars, and/or hormone and sugar signaling could converge and crosstalk through specific regulatory complexes and/or metabolic enzymes. However, how sugar and hormone signals are integrated into genetic pathways that regulate the juvenile-to-adult and vegetative-to-reproductive phase transitions is still incompletely understood. Recent studies have shown that metabolic enzymes, ABA, GA and Tre6P may integrate into the miR156/*SPL*-signaling pathway. However, despite this progress, mechanistic questions remain. Future challenges include the further clarification of the antagonistic and agonistic interactions between the sugar- and hormone-derived signals in a spatio-temporal manner at the molecular level, and their link to other known important transcriptional regulatory networks.

### Conflict of interest statement

The author declares that the research was conducted in the absence of any commercial or financial relationships that could be construed as a potential conflict of interest.
